# Flower development, pollen fertility and sex expression analyses of three sexual phenotypes of *Coccinia grandis*

**DOI:** 10.1186/s12870-014-0325-0

**Published:** 2014-11-28

**Authors:** Amita G Ghadge, Kanika Karmakar, Ravi S Devani, Jayeeta Banerjee, Boominathan Mohanasundaram, Rabindra K Sinha, Sangram Sinha, Anjan K Banerjee

**Affiliations:** Indian Institute of Science Education and Research (IISER Pune), 900 NCL Innovation Park, Dr. Homi Bhabha road, Pune, 411 008 Maharashtra India; Department of Botany, Tripura University, Suryamaninagar, Tripura 799 022 India

**Keywords:** *Coccinia grandis*, Hypogynous, Epigynous, Dioecious, Gynomonoecy, Heteromorphic sex chromosomes, Sex modification, Organ identity genes, Silver nitrate

## Abstract

**Background:**

*Coccinia grandis* is a dioecious species of Cucurbitaceae having heteromorphic sex chromosomes. The chromosome constitution of male and female plants is 22 + XY and 22 + XX respectively. Y chromosome of male sex is conspicuously large and plays a decisive role in determining maleness. Sex modification has been studied in hypogynous *Silene latifolia* (Caryophyllaceae) but there is no such report in epigynous *Coccinia grandis*. Moreover, the role of organ identity genes during sex expression in *Coccinia* has not been evaluated earlier. Investigations on sexual phenotypes of *C. grandis* including a rare gynomonoecious (GyM) form and AgNO_3_ mediated sex modification have added a new dimension to the understanding of sex expression in dioecious flowering plants.

**Results:**

Morphometric analysis showed the presence of staminodes in pistillate flowers and histological study revealed the absence of carpel initials in male flowers. Though GyM plant had XX sex chromosomes, the development of stamens occurred in hermaphrodite flowers but the pollens were not fertile. Silver nitrate (AgNO_3_) application enhanced stamen growth in wild type female flowers like that of GyM plant but here also the pollens were sterile. Differential expression of *CgPI* could be involved in the development of different floral phenotypes.

**Conclusions:**

The three principle factors, Gynoecium Suppression (Su^F^), Stamen Promoting Factor (SPF) and Male Fertility (m^F^) that control sex expression in dioecious *C. grandis* assumed to be located on Y chromosome, play a decisive role in determining maleness. However, the characteristic development of stamens in hermaphrodite flowers of GyM plant having XX sex chromosomes indicates that Y-linked SPF regulatory pathway is somehow bypassed. Our experimental findings together with all other previous chromosomal and molecular cytogenetical data strongly support the view that *C. grandis* could be used as a potential model system to study sex expression in dioecious flowering plant.

**Electronic supplementary material:**

The online version of this article (doi:10.1186/s12870-014-0325-0) contains supplementary material, which is available to authorized users.

## Background

The vast majority of angiosperms are hermaphrodites having bisexual flowers and nearly 10% of the flowering plants produce unisexual flowers [1]. Sexual systems are coupled with the numerous combinations of unisexual and hermaphrodite flowers. There are about 6% angiosperms which are dioecious bearing male and female flowers on separate individuals [2,3]. Literature study suggests that dioecious plants have evolved independently and multiple times from their bisexual progenitors [4-6].

In comparison to animals, dioecious plants show relatively recent origin of sex chromosome evolution [7,8]. Sex determination in dioecious plants may be either genetically or environmentally controlled phenomenon [9]. Some dioecious plant species have fertile bisexual relatives [10], which are excellent system for sex chromosome study. The occurrence of sex chromosomes in dioecious plants is surprisingly rare and only 19 species are known to have heteromorphic sex chromosomes [10]. The heteromorphic sex chromosomes are well-studied in *Silene latifolia* (Caryophyllaceae), in which male and female plants carry XY and XX sex chromosomes respectively [11]. The Y chromosome is reported to be the largest of all chromosomes [12] and it consists of three sex determining regions viz., Gynoecium Suppression Factor (Su^F^), Stamen Promoting Factor (SPF) and Male Fertility Factor (m^F^) [13,14]. Other well-studied dioecious plants are *Rumex acetosa* exhibiting X to autosome ratio [15,16] and Poplar known for ZW system [17] for sex determination*.* In papaya, sex determination is controlled by a pair of recently evolved sex chromosomes, Y controlling male and Y^H^ controlling hermaphrodite [18]. Thus, sex chromosome study in different dioecious plant species provides an insight for better understanding of plant sex chromosome evolution.

Plant sex determination genes were so far identified from monoecious species by map based cloning approach because there is no recombination suppression at the sex determination loci [19]. Recent genomic technologies augmented the identification of X- and Y- linked genes and allowed the detection of dosage compensation of X- linked genes in *S. latifolia* [20-22]. In papaya, 8.1 Mb hermaphrodite-specific region of the Y^H^ chromosome (HSY) and its 3.5 Mb X chromosome counterpart were sequenced and annotated for identification of sex determination genes [23-25].

It is now well documented that silver nitrate (AgNO_3_) as well as silver thiosulfate (Ag_2_S_2_O_3_) have masculinizing effect on many dioecious and monoecious plants [26-29]. Beyer [30] reported that AgNO_3_ acts as an anti-ethylene agent and induces male flowers by suppressing female reproductive organs. Evidences are also there that AgNO_3_ can modify sex via inhibition of ethylene [29,31,32]. However, a study in *Silene latifolia*, contradicts this hypothesis and proposes that sex modification might be mediated by inhibition of sulfahydryl enzymes upon application of silver thiosulfate [28]. Janousek *et al*. [33] showed that 5-azacytidine treated male plants of *S. latifolia* developed hermaphrodite flowers due to hypomethylation. This indicated the possible role of epigenetic control in sex determination and modification. Another unique case of sex modification is observed due to smut fungus (*Microbotryum violaceum)* infection in *Silene latifolia*. This fungus was reported to induce the development of anthers in female flowers (XX genotype) of S*ilene latifolia* [34]. However, in this case, pollens were found sterile indicating the decisive role of Y chromosome in fertility of pollens. Investigations on sex modification in dioecious plants may enhance our knowledge on how a genetically controlled program gets modified to an altered state.

Unlike *Silene latifolia* (Caryophyllaceae)*, Rumex acetosa* (Polygonaceae), *Carica papaya* (Caricaceae), *Spinacia oleracea* (Chenopodiaceae) and *Populus* (Salicaceae) [16,17,35,36]*,* which have been well characterized to understand the mechanism of sex determination, *Coccinia grandis*, a member of Cucurbitaceae family having an inferior ovary received comparatively less attention. *Coccinia* is a small genus comprising 27 species, all dioecious in nature [37]. It is one of the few dioecious plant species, in which presence of heteromorphic sex chromosomes is reported. The chromosome constitution of male and female plants is 22 + XY and 22 + XX respectively [38]. Literature survey suggests that sexual dimorphism in *C. grandis* is determined by a large Y chromosome [38-41], which appears to be of comparatively recent origin [37]. However, the genes involved in sex determination of *C. grandis* are not yet known. Genome of *C. grandis* is almost six times smaller than that of *Silene latifolia* and is closely related to four fully sequenced genomes of Cucurbitaceae species [42,43]. Y chromosome of *C. grandis* is the largest one found in land plants; and it is heterochromatic, differently from the euchromatic Y chromosome of *S. latifolia* [43].

In addition to male and female sex forms of *C. grandis*, Kumar and Viseveshwaraiah [38] reported a gynodioecious form in which male flowers of the hermaphrodite plants were sterile. Earlier, Holstein and Renner [37] recorded a sexual phenotype of *C. intermedia* having male and female flowers/fruits on the same node. In the present investigation, we have identified a rare gynomonoecious plant (herein after referred as GyM), bearing hermaphrodite (GyM-H) and pistillate (GyM-F) flowers on the same plant. The presence of this naturally occurring GyM plant provides a great opportunity to study the genetic basis of sex determination in *C. grandis*.

To understand the floral development and sex expression in *C. grandis*, we aimed at a comprehensive characterization of sexual phenotypes through morphometric, histological, chromosomal and molecular approaches. In the present investigation, it was observed that foliar spray of AgNO_3_ is able to induce hermaphrodite flowers in wild type female plants. To determine whether Organ Identity Genes (OIGs) have any role in differentiation of the sexes, expression studies were carried out in male, female and GyM plants. To our knowledge, no such report for *C. grandis* is available in the literature.

## Results

### Morphological differences amongst three sexual phenotypes

**Figure 1:**
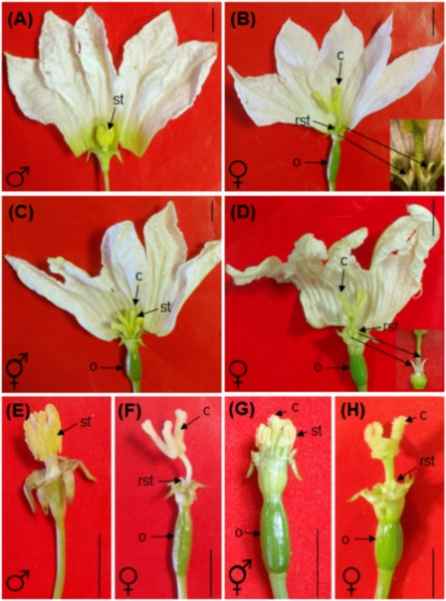
**Morphology of mature flowers of** ***Coccinia grandis*** **.** Macroscopic view of staminate flower **(A)** of male plant, pistillate flower **(B)** of female plant, hermaphrodite (GyM-H) **(C)** and pistillate (GyM-F) **(D)** flowers of gynomonoecious (GyM) plant with petals cut open. Petals removed from staminate flower **(E)** of male plant, pistillate flower **(F)** of female plant, hermaphrodite (GyM-H) **(G)** and pistillate (GyM-F) **(H)** flowers of gynomonoecious (GyM) plant to show inner floral organs. st: Stamens, c: carpels, rst: rudimentary stamens, o: ovary. Scale bars =1 cm.

While there exist striking similarities in inflorescence, sepal and petal characters, differences in the morphology of mature flowers were clearly observed amongst the three sexual phenotypes. Mature male flowers were seen to be composed of three whorls having five sepals, five united petals and five (2 + 2 + 1) synandrous stamens (Figure 1A,E). In contrast, the female flowers were composed of four whorls. While sepals and petals were identical to male flowers, the stamens were found to be arrested as rudimentary staminodes. The gynoecium consisted of three carpels having a fused style with three bifid stigmas (Figure 1B,F). The GyM plants bear two different types of flowers (i) hermaphrodite (GyM-H) and (ii) pistillate (GyM-F) (Additional file 1: Figure S1). The GyM-H flowers had four whorls, almost similar to the flowers of female sex; the only difference being here that the staminodes gradually developed to mature stamens (Figure 1C,G). It was also observed that some of the GyM-H flowers exhibited incomplete growth of stamens (Additional file 2: Figure S2A) as well as petaloid stamens (Additional file 2: Figure S2C). The organization of floral organs in GyM-F flowers of GyM plant was found to be similar to that of wild type female plant (Figure 1D,H). We observed random positional distribution of GyM-F and GyM-H flowers in GyM plant and the ratio of these flowers was found to be approximately 30:70 during the months of April to July. The phylogenetic analysis using *mat*K and *trn*S^GCU^-*trn*G^UCC^ intergenic spacer region, revealed that the GyM plant is another sexual phenotype of *Coccinia grandis* (Additional file 3: Figure S3). Except for the three sexual phenotypes of *Coccinia grandis* (Additional file 4: Table S1)*,* sequences for constructing the phylogenetic tree were used from the previously published data [37]. Seed content of fruits from female plant (seed number and seed weight per fruit) was observed to be higher than that of fruits from GyM plant (Additional file 5: Figure S4A,B).

### Histological analysis

**Figure 2:**
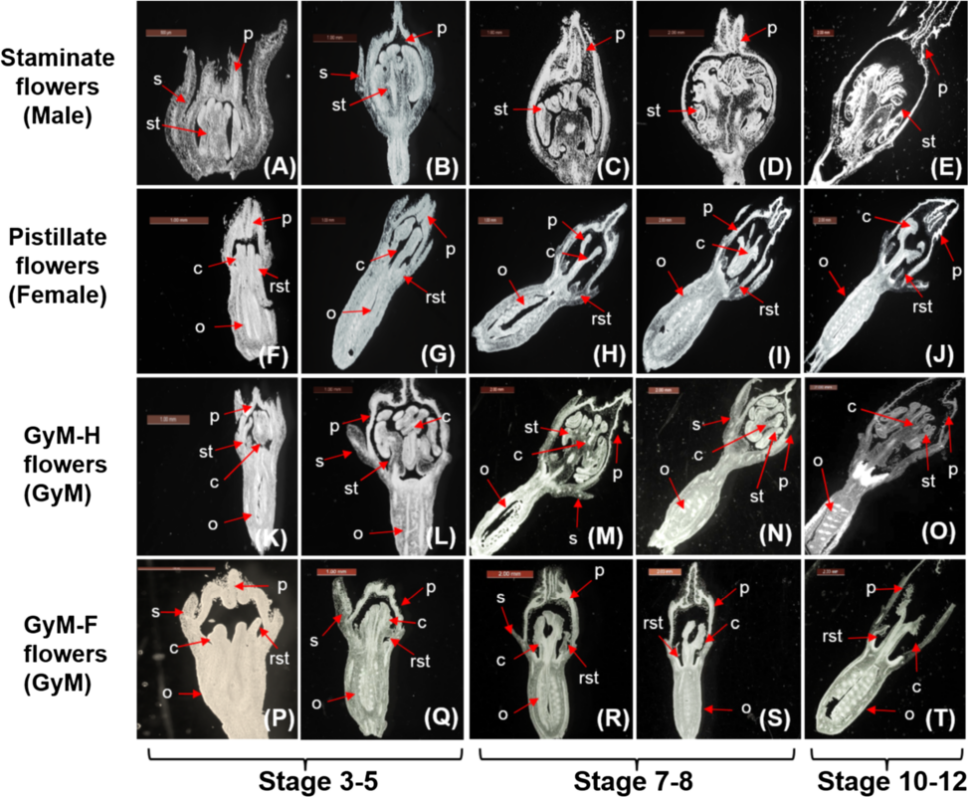
**Longitudinal sections (L.S) of flower buds at different developmental stages. (A–E)** are the sections of staminate flowers of male plant, **(F–J)** are the sections of pistillate flowers of female plant, **(K–O)** and **(P–T)** are the sections of hermaphrodite (GyM-H) and pistillate (GyM-F) flowers of gynomonoecious (GyM) plant respectively. p: Petals, s: sepals, c: carpels, st: stamens, rst: rudimentary stamens, o: ovary. Scale bars are 500 μm in A, 1 mm in B, C, F, G, H, K, L, P and Q, and 2 mm in D, E, I, J, M, N, O, R, S and T.

To understand the sequential development of sex organs, histological analysis was carried out at different stages of flower development for all three sexual phenotypes (Figure 2A–T).

Male: Histological observation of male flowers (stages 3–4, Additional file 6: Figure S5A) showed the presence of sepals, petals and stamens having no sign of carpel initials (Figure 2A). Even in the later stages of flower development, any rudimentary carpel was not observed. However, the possibility of presence of carpel initials in primordial stages of flower development cannot be completely ruled out. Further growth of stamens was observed in the successive stages of male flower development (Figure 2B–D). Finally, in stage 12 (Additional file 6: Figure S5A), mature pollens were found inside the anthers when petals were about to open (Figure 2E, Additional file 7: Figure S6).

Female: Whereas female flowers (stages 3–4, Additional file 6: Figure S5B) exhibited the presence of sepals, petals, stamen initials and carpels having an inferior ovary in four whorls (Figure 2F). While development of the androecium remained arrested in early stages, growth of the gynoecium was noted in successive stages of development (Figure 2G–I). At stage 12 (Additional file 6: Figure S5B), when the petals were about to open, the gynoecium was found to be completely developed (Figure 2J).

GyM: Presence of sepals, petals, stamens and carpel initials along with an inferior ovary was observed in four successive whorls of GyM-H flowers at early stages of development (Figure 2K, Additional file 2: Figure S2B). Further growth of the gynoecium and androecium occurred in successive stages of development (Figure 2L–N) and at stage 12 (Additional file 6: Figure S5C), growth of the gynoecium and androecium was found to be complete (Figure 2O). However, development of GyM-F flowers in GyM plant was found to be identical to that of wild type female plant (Figure 2P–T).

### Chromosomal study

**Figure 3:**
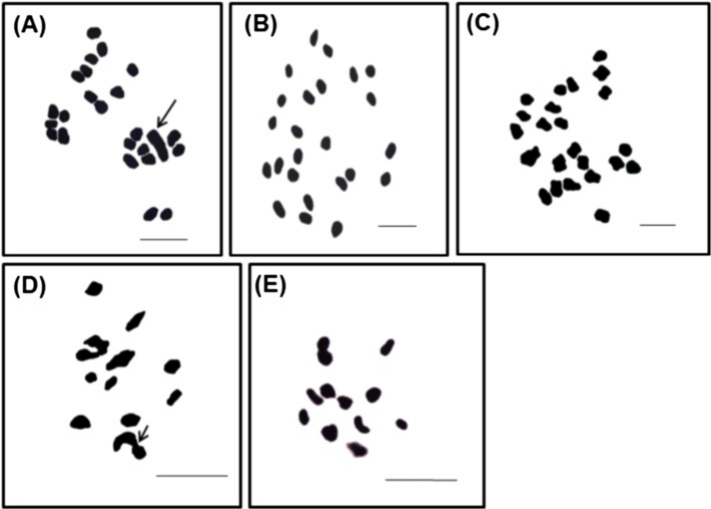
**Metaphase chromosomes of** ***C. grandis*** **.** Mitotic metaphase chromosomes showing 2*n* =24 chromosomes of male **(A)** (arrow indicates the large Y chromosome), female **(B)** and gynomonoecious (GyM) **(C)** plants. Meiotic metaphase chromosomes showing 12 bivalents of male **(D)** (arrow indicates end to end pairing of X and Y chromosomes), gynomonoecious (GyM) **(E)** plants. Scale bar =5 μm.

In order to have a better understanding of the relation between male, female and GyM plants of *C. grandis* growing in the same environment, comparative cytological studies were carried out. The somatic chromosome number of male, female and GyM plant was found to be 2*n* = 24 (Table 1). Sex chromosomes were heteromorphic and in male plants Y chromosome was conspicuously large (Figure 3A). In wild type female and GyM plants, the chromosome constitution is 22 + XX (Figure 3B,C). The karyotype of wild type female and GyM plant showed similarity to a considerable extent (Figure 3B,C). Meiotic studies of male sex showed end to end pairing between X and Y chromosomes (Figure 3D). In contrast, normal pairing of homologous chromosomes were found in GyM-H flowers of GyM plant (Figure 3E).

**Table 1:** **Numerical data on somatic chromosome complements of** ***C. grandis*** **(male, female and gynomonoecious (GyM) plants)**

**Chromosome numbers**	Chromosome size (μm)* (Mean ± SD)			**F%**			**Position of centromere**		
	**Male**	**Female**	**GyM**	**Male**	**Female**	**GyM**	**Male**	**Female**	**GyM**
1	1.92 ± 0.07	2.01 ± 0.03	1.92 ± 0.06	50	50	50	m	m	m
2	1.92 ± 0.06	1.92 ± 0.06	1.92 ± 0.07	45	45	48	nm	nm	nm
3	1.76 ± 0.03	1.84 ± 0.06	1.82 ± 0.04	50	50	50	m	m	m
4	1.76 ± 0.03	1.76 ± 0.03	1.76 ± 0.06	45	47	45	nm	nm	nm
5	1.62 ± 0.07	1.62 ± 0.03	1.65 ± 0.03	33	33	33	sm	sm	sm
6	1.62 ± 0.03	1.62 ± 0.03	1.65 ± 0.03	46	46	46	nm	nm	nm
7	1.54 ± 0.09	1.54 ± 0.07	1.54 ± 0.07	43	43	43	nm	nm	nm
8	1.54 ± 0.03	1.54 ± 0.07	1.54 ± 0.09	46	47	47	nm	nm	nm
9	1.40 ± 0.02	1.54 ± 0.07	1.43 ± 0.05	44	47	47	nm	nm	nm
10	1.22 ± 0.09	1.22 ± 0.05	1.23 ± 0.03	44	46	46	nm	nm	nm
11	1.22 ± 0.01	1.22 ± 0.05	1.23 ± 0.03	45	45	46	nm	nm	nm
12	1.10 ± 0.06	1.10 ± 0.06	1.10 ± 0.06	44	46	47	nm	nm	nm
Y^1^	4.60 ± 0.07	-	-	48	-	-	nm	-	-

*Mean of 5 metaphase plates. GyM: gynomonoecious, m: metacentric, nm: nearly metacentric, sm: submetacentric. The karyotype of male and female plants was compared with the gynomonoecious (GyM) chromosomes. Y^1^: Single Y chromosome present in male sex.

### AgNO_3_ induced sex modification

**Figure 4:**
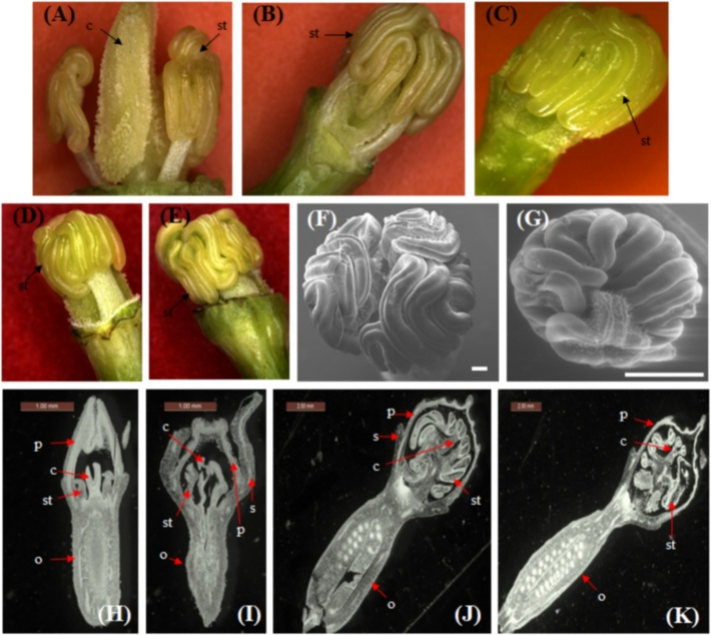
**Effects of silver nitrate (AgNO**_**3**_ **) solution on female plant. (A-C)** are the pictures of female flowers after spraying of AgNO_3_ solution showing gradual enhanced stamen growth. Magnified view of stamens in **(D)** pistillate flowers of AgNO_3_ treated female plant and **(E)** hermaphrodite (GyM-H) flowers of gynomonoecious (GyM) plants. Scanning electron micrographs of top view of **(F)** pistillate flowers from AgNO_3_ treated female plant and **(G)** hermaphrodite (GyM–H) flowers of gynomonoecious (GyM) plants. Petals and sepals have been removed to better view sexual structures. Longitudinal sections **(H-K)** of flower buds of silver nitrate treated female plant (after spraying of 35 mM silver nitrate solution). H, I – flower buds of stage 5, J – flower bud of stage 8 and K – flower bud of stage 10. p: Petals, s: sepals, c: carpels, st: stamens, o: ovary. Scale bars are 300 μm in F, 1 mm in G, H and I, and 2 mm in J and K.

Different concentrations of silver nitrate (AgNO_3_) solution were sprayed on the basal leaves of male, female and GyM plant (Additional file 8: Table S2). Newly emerging flower buds of wild type female plants showed enhanced growth of stamens after application of AgNO_3_ solution (Figure 4A–D) whereas; male flowers did not show any changes in floral structure. Histological studies further confirmed the dose dependent stamen growth in wild type female flowers (Figure 4H–K; Additional file 8: Table S2). However, concentrations higher than 35 mM had lethal effect. At dosages of 30 and 35 mM of AgNO_3_, the morphology of newly developed flowers was comparable to GyM-H flowers after 10-12 days of observation (Figure 4D–G). Interestingly, all mature flowers in GyM plant were found to be hermaphroditic after application of AgNO_3,_ indicating that even the staminodes of pistillate flower buds have developed into mature stamens (Additional file 9: Figure S7).

### Mating experiments and pollen fertility

Mating experiments were designed to investigate the fertility of pollens from male flowers and GyM-H flowers (Table 2). The crosses between male and emasculated GyM-H resulted in 83.33% of fruit setting. No fruit setting was recorded in crosses between GyM-H and wild type female flowers. It was also noted that 90% fruit setting occurred in crosses between male and wild type female (Table 2). Similarly, the crosses between the wild type male and the pistillate flowers of GyM plant also yielded 93% of fruit setting. However, no fruit setting was achieved in crosses between GyM-H and GyM-F flowers and by selfing GyM-H (Table 2).

**Table 2:** **Mating design and percentage of fruit set in** ***C. grandis***

**Mating design**	**Pollen source**	**No. of fruit set**	**% fruit set**	**Remarks**
Male X GMH (emasculated)	Male	8.33 ± 0.577	83.33	Fertile pollen
Male X GMF	Male	9.33 ± 0.577	93.33	Fertile pollen
Male X Female	Male	9.0 ± 1.00	90.00	Fertile pollen
GMH self	GMH	0.00	0.00	Sterile pollen
GMH X GMF	GMH	0.00	0.00	Sterile pollen

Replications =3, N =30, No. of crosses/ mating design are 10 for all the above sets.

GyM-H: hermaphrodite flower from gynomonoecious (GyM) plant, GyM-F: pistillate flower from gynomonoecious (GyM) plant.

**Figure 5:**
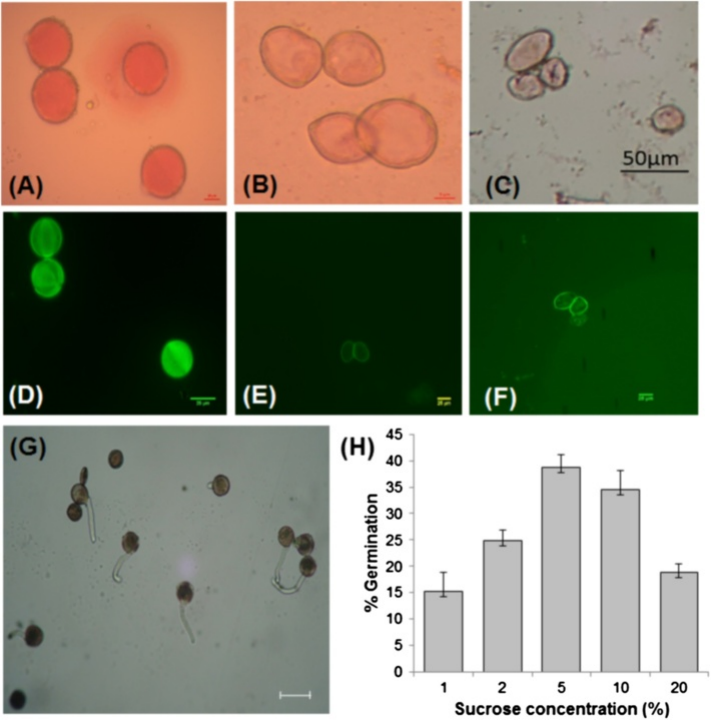
**Viability tests of pollens from male, gynomonoecious (GyM) and AgNO**_**3**_ **treated female plants.** Pollens stained with 1% acetocarmine from male **(A)**, gynomonoecious (GyM) **(B)** and AgNO_3_ treated female **(C)** plants. **(D)**, **(E)** and **(F)** are the fluorescein diacetate (FDA) stained pollens from male, gynomonoecious (GyM) and AgNO_3_ treated female plants respectively. Pollens stained with acetocarmine **(A)** and FDA **(D)** are viable. Scale bars are 10 μm in A, 5 μm in B, 50 μm C, and 25 μm in D, E and F. **(G)** Highest germination of male pollens in 5% sucrose solution. Scale bar =50 μm. **(H)** Graphical representation of the germination percentage in different concentrations of sucrose solutions. Means ± standard errors are reported in the graph; n = 10.

For viability assays, pollens were isolated from opened flowers of male, GyM-H and converted flowers of AgNO_3_ treated female plant. Pollens from male flowers took aceto-carmine stain; whereas pollens from GyM-H flowers and converted flowers of AgNO_3_ treated female plant did not retain any stain (Figure 5A–C). These results were reconfirmed with FDA test (Figure 5D–F). In addition, pollen germination was also tested for male, GyM plant and AgNO_3_ treated female plant. Highest frequency of pollen germination (38%) was achieved when pollens of male flowers were incubated in 5% sucrose solution containing required amount of Ca(NO_3_)_2_ and H_3_BO_3_ (Figure 5G,H). In contrast, pollens of hermaphrodite flowers of GyM and AgNO_3_ treated female plant did not show any germination when incubated in different germinating media. From the above results, we concluded that pollens of male flowers are fertile and pollens from GyM-H and converted flowers of AgNO_3_ treated female plant are sterile in nature.

### Identification and expression analysis of Organ Identity Genes (OIGs)

**Figure 6:**
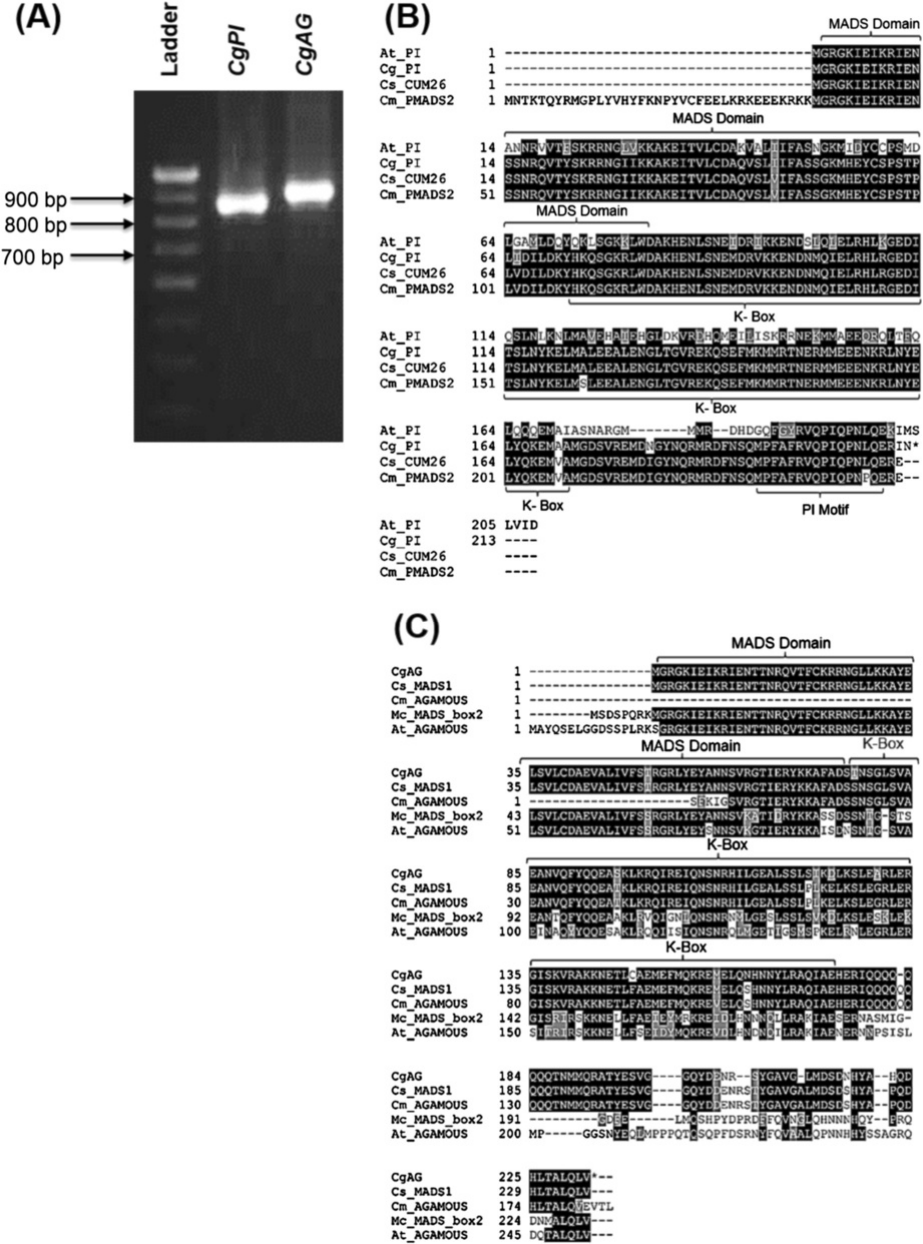
**Full length** ***CgPI*** **and** ***CgAG*** **transcript isolation and multiple sequence alignment of deduced amino acid sequences. (A)** Amplification of full length *CgPI* and *CgAG* transcripts from total RNA harvested from flower buds. **(B)** Comparison of CgPI with other PIISTILLATA-like genes. **(C)** Comparison of CgAG with other AGAMOUS-like genes. Conserved regions are shaded in black. *At_PI*, *Cg_PI*, *Cs_CUM26* and *Cm_pMADS2* are *PISTILLATA* like genes from *Arabidopsis thaliana, Coccinia grandis, Cucumis sativus* and *Cucumis melo* respectively*. Cg_AG*, *Cs_MADS1*, *Cm_AGAMOUS*, *Mc_MADS_box2*, *At_AGAMOUS* are *AGAMOUS* like genes from *Coccinia grandis*, *Cucumis sativus*, *Cucumis melo*, *Momordica charantia* and *Arabidopsis thaliana* respectively. MADS domain and K-box are identified by NCBI’s conserved domain database and marked accordingly.

In order to understand whether B and C class Organ Identity Genes (OIGs) have any role in determining the sex of the developing flowers of male, female and GyM plant, *CgPI* (a B class OIG) and *CgAG* (a C class OIG) were isolated and an expression analysis was carried out using quantitative real-time PCR (qRT-PCR). The degenerate primers based on the conserved amino acid sequences of PI (*PISTILLATA)* and AG (*AGAMOUS),* yielded ~350 bp of *PISTILLATA* (*CgPI*) and ~250 bp of *AGAMOUS* (*CgAG*) homologs through RT-PCR reaction. The partial sequences for *CgPI* [DDBJ:AB859715] and *CgAG* [DDBJ:AB859714] have been deposited in DDBJ. Full length transcript sequences were deduced from 5′ and 3′ RACE products and amplicons of *CgPI* (~893 bp) and *CgAG* (~952 bp) were obtained (Figure 6A). cDNA for *CgPI* and *CgAG* coded for putative proteins of 212 and 232 amino acids respectively. The deduced amino acids sequences for both the genes showed high conservation when aligned with other *PISTILLATA* and *AGAMOUS* like genes (Figure 6B,C). Two consensus regions, MADS domain and K-box were found on the deduced amino acid sequences (Figure 6B,C).

**Figure 7:**
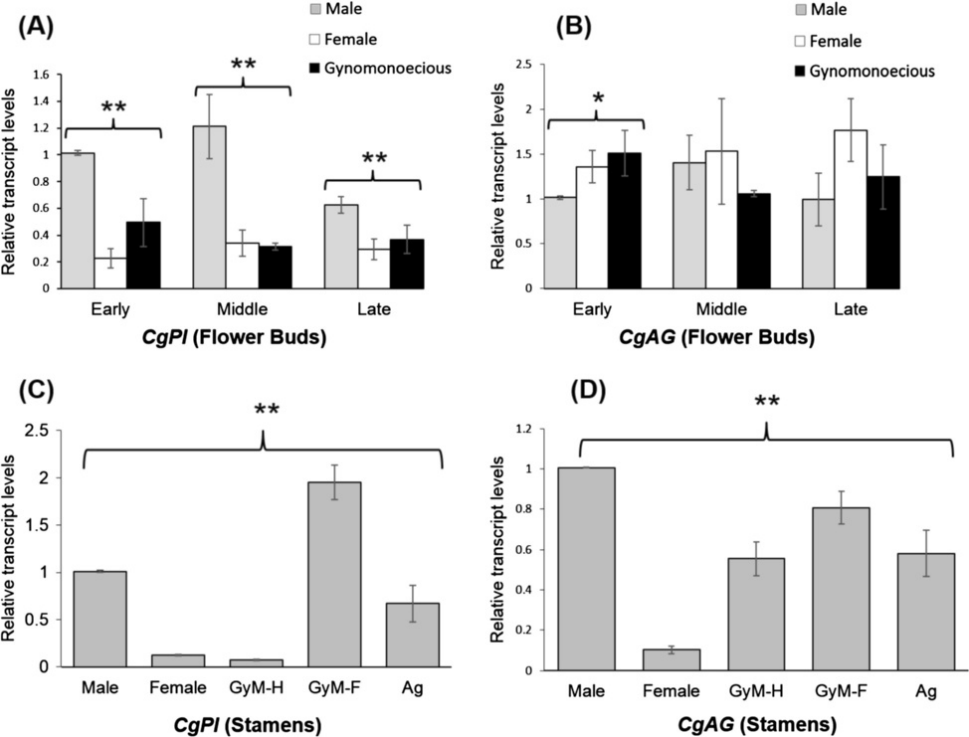
**Expression analyses of Organ Identity Genes (OIGs) from** ***C. grandis.*** Expression patterns of *CgPI* **(A)** and *CgAG* **(B)** in flower buds of male, female and gynomonoecious (GyM) *C. grandis* at different developmental stages (early, middle and late) by quantitative real time PCR (qRT-PCR). Stamen specific expression patterns of *CgPI* **(C)** and *CgAG* **(D)** from flowers (late developmental stage) of male, female (rudimentary), hermaphrodite (GyM-H) and pistillate (GyM-F, rudimentary) flowers of gynomonoecious (GyM) and converted flowers of AgNO_3_ treated plants. Error bars indicate SD (standard deviation) of three biological replicates each with three technical replicates. Asterisks indicate statistical differences as determined using single factor ANOVA (*P <0.05 and **P <0.01). Early: from 3^rd^ to 5^th^ stages, middle: 6^th^ to 8^th^ stages, late: 9^th^ to 12^th^ stages.

*CgPI*, a B class gene required for petal and stamen development, was found to be expressed in male, wild type female and GyM flower buds (Figure 7A). Expression of *CgAG*, a C class gene essential for stamen and carpel development, was also noted in male, wild type female and GyM flower buds (Figure 7B). Our results showed that both these genes are expressed in all developmental stages (Additional file 6: Figure S5) (early, middle and late) of flowers from male, female and GyM plant. *CgPI* had a significant difference of expression across all three sexual forms during early, middle and late developmental stages (Figure 7A), while *CgAG* showed significant differential expression in buds of early stages only (Figure 7B). We have also noted that *CgPI* expression is comparatively high in male flower buds than that of wild type female buds. However, GyM flowers exhibited an intermediate level of *CgPI* expression in early and late staged buds (Figure 7A). Further, our results for stamen-specific expression analysis showed a significant difference for both *CgPI* and *CgAG* levels between stamens of male, GyM-H, AgNO_3_ treated female plant, rudimentary stamens of GyM-F and wild type female plant (Figure 7C,D). Surprisingly, rudimentary stamens of GyM-F showed higher *CgPI* expression than stamens of GyM-H flowers (Figure 7C).

## Discussion

### Carpel and stamen differentiation programmes follow independent pathway

In contrast to *Silene latifolia,* where rudimentary gynoecium is found in male flower [44,45] histological study revealed the absence of carpel initials even at early stage of development (stages 3-4, Additional file 6: Figure S5A) of male flower in *C. grandis* (Figure 2A,B). Though stamen initiation occurs in female plants, its growth is arrested at early stages (stages 4–5, Additional file 6: Figure S5B) of flower development (Figure 2G–I) leading to the retention of sterile staminode in mature flower. This indicates a functional interference in the male differentiation pathway of female flowers as was reported in *Silene latifolia* [14]. In GyM-H flowers, androecium and gynoecium develop simultaneously till maturation (Figure 2K, L) and arrest of stamen or carpel growth is not observed (Figure 2M–O). However, in pistillate flowers of GyM plant, arrest of stamen growth occurs at early stages like the flowers of wild type female plant (Figure 2Q,R). The development of mature carpel with arrested stamen growth as evidenced by the presence of rudimentary staminodes in pistillate (GyM-F) flowers and the synandrous stamens with fully grown carpel in GyM-H flowers indicate that the carpel and stamen differentiation programmes follow independent pathway.

### Gynomonoecious (GyM) *C. grandis -* is not a Y-deletion mutant

While investigating the morphological differences between male and female sexes, we have recorded the existence of a GyM plant in the north eastern part of India (Tripura) that exhibited morphological characteristics similar to that of male and female sex forms of *C. grandis.* The morphological characterization and the phylogenetic analysis, based on the tree constructed with *mat*K and *trn*S^GCU^-*trn*G^UCC^ intergenic spacer regions clearly establish the identity of the GyM plant to be another sexual phenotype of *C. grandis*.

The present record of diploid chromosome number 2*n* = 24 in both male and female sexes (Figure 3A,B) and the presence of heteromorphic sex chromosomes in male plants corroborate previous findings and validate XY sex determination system [38,43,46-48]. The characteristic end to end pairing between X and Y chromosomes (Figure 3D) indicates recombination between Pseudo Autosomal Region (PAR) [43] and that there are non-recombining regions between X and Y chromosomes as was suggested by other researchers to explain the genetic basis of sex determination in some dioecious plants [13,49]. The absence of carpel initial in male plant suggests that the Y chromosome has a dominant gynoecium suppressor gene at the non-recombining region like that of *S. latifolia* [13]. The karyotype of GyM plant shows high degree of similarity to that of wild type female (Table 1; Figure 3B,C). The smallest bivalent found in metaphase I of hermaphrodite flower does not match with the size of X chromosome of heteromorphic pair found in male sex (Figure 3D,E). Therefore, it requires further test to assume the smallest chromosomes as X chromosome [43] and at this stage, it remains inconclusive due to the unavailability of X- specific probes in *C. grandis*. The absence of male specific Y chromosome in GyM plant and normal pairing between homologous chromosomes (Figure 3C,E) indicate that GyM plant also possesses 22 + XX chromosomes and contains genetic information necessary to produce both pistillate and hermaphrodite flowers. The lability of the expression of hermaphroditism suggests that the GyM plant is genotypically a female individual and not a Y- deletion mutant. The questions that might arise are firstly, in absence of the Y chromosome, how does the development of stamens occur in hermaphrodite (GyM-H) flowers of GyM plant? And secondly, what factors contribute to the development of hermaphrodite (GyM-H) and pistillate (GyM-F) flowers in the same plant?

### Factors stimulating stamen development in GyM plant in absence of Y chromosome

In contrast to fertile and viable pollens of male flowers (Figure 5A,D), pollens of GyM-H flowers are sterile in nature and remain immature even at later stages of development (Figure 5B,E). The results of breeding experiments negate the possibility of self-fertilization and thus fruit setting occurs only through allogamy or cross pollination when pollens from male sex act as donor (Table 2). This indicates that viable and fertile pollens are produced in male plants only and that male fertility factor is located on Y chromosome. Evidently, male fertility is controlled by the Y chromosome and it plays a decisive role in determining sex in *C. grandis* [50]. Similar to *Silene latifolia* [13,14], our experimental results suggest that in *C. grandis*, at least three key factors: Gynoecium Suppressor, Stamen Promoting Factor And Male Fertility Factor have assembled and possibly rearranged during evolution of the Y chromosome. However, development of the stamen with sterile pollens in GyM-H flowers of genotypically female GyM plant suggests that the factors stimulating stamen development might be present elsewhere in the genome, while the male fertility factor may be absent. Scutt *et al*. [51] reported that infected female *S. latifolia* with XX sex chromosomes can develop morphologically normal stamens. Whereas, Farbos *et al*. [14] have shown that in *Silene latifolia,* Gynoecium Suppression Factor (GSF/Su^F^) and Stamen Promoting Factor (SPF) regions of Y-chromosome behave as linked dominant traits and that SPF is absent in female plants with XX sex chromosomes. In the absence of Y or truncated Y chromosome, the mechanism of stamen development in GyM-H flower of *C. grandis* is not clear but needs further investigation.

### Phenomenon of silver nitrate induced stamen development resembles that of GyM plant

Silver nitrate stimulated stamen development in female plants of *C. grandis* mimics the pathway of stamen development in GyM plant (Figure 4D–G). Y chromosome is absent in both of these sexual phenotypes and pollens of converted flowers of AgNO_3_ treated female plant are sterile in nature like the pollens of GyM-H flowers (Figure 5C,F). This suggests that stamen development is induced in wild type female by an unknown pathway which is independent of Y-mediated mechanism as was reported in *Silene latifolia* [28]. But there is no clue how this signal is transmitted from leaves to flowers that leads to sex modification. However, silver nitrate effect is transient and normal female flower develops after a period of 15-20 days. This may be due to the fact that the effective AgNO_3_ concentration below the threshold level cannot impede the molecular mechanism leading to the formation of gynoecium with arrested stamen growth. It appears that AgNO_3_ at an optimum concentration stimulates stamen development in wild type female and GyM-F of *C. grandis* possibly by a temporary delay in functional interference of male differentiation pathway. In such a condition, possibility of the presence of male repressive factor in untreated plants and its de-repression by AgNO_3_ molecule in treated wild type female and GyM-F cannot be ruled out.

### Differential expression of OIGs and the development of the three different floral phenotypes

The B and C function genes viz. *CgPI* and *CgAG* show homology to *CUM26* and *MADS1* of *Cucumis sativus* respectively. qRT-PCR studies suggest that like *Silene latifolia* [52], the male flowers of *C. grandis* had higher *CgPI* expression compared to wild type female flowers (Figure 7A). This observation was also true for stamen specific expression analysis (Figure 7C). The high expression of *CgPI* in stamens of GyM-F and reduced expression in stamens of GyM-H flowers cannot be explained currently and would require future investigations. This study indicates that OIGs might be under differential regulation in male, female and GyM plant leading to the development of male, wild-type female and GyM-H as well as GyM-F flowers. To this effect, further studies are required to understand the role of *ACS* (aminocyclopropane-1-carboxylate synthase) and *WIP1* (Wound Inducible Protein 1) genes, which were shown to govern sex expression in a related species, *Cucumis melo* [53,54]. The sex-determining locus A of melon encodes an ethylene biosynthesis enzyme, CmACS-7, that represses stamen development in female flowers. The G locus of melon encodes *CmWIP1*, a transcription factor that represses carpel development in male flowers. Also, it has been shown that the role of *ACS* gene is conserved in another member of the family *Cucumis sativus* [55]. Future investigation on functional validation of these genes would be necessary to decipher their role in sex expression and modification.

## Conclusions

There is no doubt that the ‘female suppressing’ functions of the Y chromosome in male *C. grandis* is an initial event towards the establishment of the sexual dimorphism. The process of stamen initiation occurs in wild type female and GyM plants even in the absence of Y chromosome but the arrest of further development of stamens suggests a possible interference in ‘Stamen Promoting’ functions (SPF). The pollens of GyM-H and converted flowers of AgNO_3_ treated female plants were sterile indicating that the male fertility factor is located on Y chromosome which is solely responsible for pollen fertility. The significance of GyM plant of *C. grandis* lies in its ability to develop stamens with sterile pollens because such evidences were not reported in any other plants including gynomonoecious *Silene* species [56,57]. The characteristic development of stamens in hermaphrodite flowers of GyM having XX sex chromosomes and AgNO_3_ modified wild type female flowers is mediated by an unknown mechanism bypassing the Y-linked SPF regulatory pathway. Our experimental findings together with all other previous chromosomal and molecular cytogenetical data strongly support the view that *C. grandis* could be used as a potential model system to study sex expression in dioecious flowering plant.

## Methods

### Plant material and stages of flower development

Tuberous roots of wild type male, female and GyM *Coccinia grandis* were collected from west Tripura and grown in the experimental fields of IISER Pune and Tripura University (Herbarium voucher for gynomonoecious *C. grandis* is provided in Additional file 10: Figure S8). The clones were maintained in the experimental plots since last two years. Leaves and flowers from male, female and GyM plants were harvested periodically and were frozen in liquid nitrogen for various experimental purposes. Based on the size of the flower buds, we have divided the process of flower initiation into 12 different stages in ascending order. Out of the 12 different stages, first two stages were studied under stereomicroscope and only the flower buds from 3^rd^ to 12^th^ stages (Additional file 6: Figure S5) were considered for stage specific histological study. For qRT-PCR expression analyses, flower buds were grouped into three different categories viz., early (from 3^rd^ to 5^th^ stages), middle (6^th^ to 8^th^ stages) and late (9^th^ to 12^th^ stages) for experimental purposes. In addition, stamens were also harvested from the flowers (late developmental stage) of male, GyM-H, and converted flowers of AgNO_3_ treated female plant as well as rudimentary stamens of wild type female and GyM-F flowers for expression analysis.

### Histology of flower buds

To understand the patterns of flower development of male, female and GyM plants, flower buds of different stages (Additional file 6: Figure S5) were harvested and fixed in 1:3 acetic acid-ethanol solution and kept at 4°C for overnight. Longitudinal sections (L.S.) of flower buds of different developmental stages were prepared as described by Cai and Lashbrook [58] with the following modifications. The fixed tissue was dehydrated with 75% ethanol for 40 min, 95% ethanol for 40 min and finally washed thrice with 100% ethanol, each with 45 min intervals. The material was then treated with 50-50% ethanol-xylene for 45 min, followed by clearing with 100% xylene for 45 min. Xylene was replaced with paraplast wax at 59°C. Then the tissue was embedded in the paraplast blocks. Thin paraplast sections (10 μm) were mounted on the slides with water at 50°C. The wax was cleared from the slides by washing with 100% xylene. The images of the cleared slides were finally documented in Leica MZ 16 FA microscope.

### Analysis of mitosis and meiosis chromosomes

To analyze mitotic and meiotic chromosomes, investigations were carried out through modified aceto-orcein and aceto-carmine staining techniques [59]. Young leaf-tips were pre-treated in saturated solution of para-dichlorobenzene for 5 h at 10–15°C followed by overnight fixation in 1:3 acetic acid-ethanol mixture. The fixed leaf tips were then hydrolyzed with 5 N HCl at 10°C for 15 min, stained with 2% aceto-orcein for overnight and finally squashed in 45% acetic acid. For meiotic chromosome preparation, young flower buds were fixed in 1:3 acetic acid-ethanol mixture for 2–3 h followed by 45% acetic acid treatment for 30 min. Suitable anthers were smeared with 1% aceotocarmine stain and metaphase I stages of wild type male and GyM-H flowers were documented. Photomicrographs were taken with Nikon Eclipse E200 Microscope using Sony Cybershot DSC-W320 Camera (digitalized with Optical Zoom - ×4, 14.1 megapixels) with ×10 eye-piece and ×100 oil immersion lens. Each photograph was suitably enlarged and digitally processed under horizontal and vertical resolution at 350 dpi for male and female mitotic metaphase chromosomes and at 72 dpi for GyM mitotic chromosomes. Meiotic metaphase chromosomes of male and GyM-H flower bud were also processed at 350 dpi for better resolution.

### Mating design and fruit set analysis

To test the fertility of pollens from male and GyM plants of *C. grandis*, four controlled cross experiments and one self-pollination experiment were designed (Table 2). GyM-H flowers were emasculated before mating. Ten flowers were bagged for each experimental set. The cotton cloth bag was removed after 7 days of controlled pollination and observation was made to each of the flowers. All experimental sets were repeated thrice.

### Pollen germination and viability test

To determine the germination rate, fresh pollens from mature male and GyM-H flowers were incubated in germination media with different concentrations of sucrose (1%, 2%, 5%, 10% and 20%) containing 2 mM Ca(NO_3_)_2_ and 2 mM H_3_BO_3_ [60]. Germination was scored after 1 h of incubation at room temperature. In order to check the fertility of pollens from mature flowers of male plant, GyM-H and converted flowers of AgNO_3_ treated female plant; pollen grains of each kind were further stained with 1% aceto-carmine solution for 5 min and were documented under the light microscope. Fluorescein diacetate (FDA) test was performed to check the viability of pollens according to the protocol as described by Heslop-Harrison and Heslop-Harrison [61].

### Identification and isolation of full length *AGAMOUS (CgAG)* and *PISTILLATA (CgPI)* homologs

To isolate AGAMOUS (*CgAG*) and PISTILLATA (*CgPI*) homologs from *C. grandis*, total RNA was isolated from harvested flower buds and pooled from all three sexual forms. Degenerate primers (Additional file 11: Table S3) were designed from conserved sequences of PI and AG homologs (Additional file 12: Table S4) using iCODEHOP [62]. Approximately, 2 μg of total RNA was used for RT-PCR reactions using SuperScript^®^ III One-Step RT-PCR System with Platinum^®^Taq (Invitrogen-12574-018). The first step of reaction included incubation at 50°C for 20 min for cDNA synthesis followed by 94°C for 2 min, 40 cycles of incubations at 94°C for 15 s, 50°C for 30 s and 68°C for 35 s. Final extension was carried out at 68°C for 5 min. Amplified products were resolved on 2% agarose gel and cloned into pGEMT vector and finally sequence verified. These sequences were used to design primers for 5′ and 3′ RACE to obtain the full length transcript sequences (Additional file 11: Table S3). RACE ready cDNAs were generated using SMARTer RACE cDNA synthesis kit (Clontech). 5′ and 3′ sequences were further amplified from the cDNAs using the designed primers and the universal primer provided with the kit. Amplified 5′ and 3′ regions of *CgPI* and *CgAG* were sequence verified. Primers were designed to amplify full length transcripts. Deduced amino acid sequences were aligned with other PISTILLATA and AGAMOUS like genes using Clustal Omega and consensus sequences were shaded using Boxshade server [55]. Conserved domains were identified using NCBI’s Conserved Domain Database (CDD) search [63].

### Quantitative real time PCR (qRT-PCR) analysis

For expression analysis, qRT-PCR was carried out using RNA extracted from whole flower buds at each of the three stages (early, middle and late). RNA was also extracted from the stamens of flowers (late developmental stage) of male, female, GyM-H, GyM-F and converted flowers of AgNO_3_ treated female plant using the RNeasy Plant Mini Kit (Qiagen) as per the manufacturer’s instructions. The yield and RNA purity was determined using Nanodrop 2000c Spectrophotometer (Thermo Scientific, Wilmington, USA) and visualized by gel electrophoresis. Two hundred nanograms (200 ng) of total RNA was used for complementary DNA (cDNA) synthesis by SuperScript III reverse transcriptase (Invitrogen) using an oligo(dT) primer for *CgPI* and *CgAG* genes. 18S rRNA gene was used for normalization for all the reactions. For 18S, fifty nanograms (50 ng) of total RNA was used for complementary DNA (cDNA) synthesis using gene specific reverse primer (Additional file 11: Table S3). qRT-PCR was performed on Roche LightCycler 96 with gene specific forward and reverse primers (Additional file 11: Table S3). The reactions were carried out using KAPA SYBR green master mix (Kapa Biosystems) and incubated at 95°C for 5 min followed by 40 cycles of 95°C for 10 s and 60°C for 20 s. PCR specificity was checked by melting curve analysis, and data were analyzed using the 2^–∆∆CT^ method [64].

### Foliar spray of AgNO_3_ in female and GyM plants

In order to assess the AgNO_3_ effect, different concentrations of AgNO_3_ solutions (20 mM, 25 mM, 30 mM, 35 mM and 40 mM) were periodically sprayed on the leaves of female and GyM plants (Additional file 8: Table S2) prior to flowering stage. After 12 days of foliar spray of 35 mM AgNO_3_ solution, the converted flower buds were harvested at different stages and fixed in 1:3 acetic acid- ethanol mixture for stage specific histological study.

All the supporting data are included as additional files only.

## Additional files

Additional file 1: Figure S1. Floral phenotypes on gynomonoecious (GyM) plant. GyM plant showing both hermaphrodite (GyM–H) and pistillate (GyM–F) flowers on the same twig. Additional file 2: Figure S2. Morphology of hermaphrodite (GyM–H) flowers of gynomonoecious (GyM) plant. Mature hermaphrodite (GyM-H) flower of gynomonoecious (GyM) showing incomplete development of stamens (A) and petaloid stamens (C). Longitudinal sections of early developmental stage of hermaphrodite (GyM–H) flower of gynomonoecious (GyM) plant (B). p: petals, s: sepals, c: carpels, st: stamens, rst: rudimentary stamens, o: ovary, pst: petaloid stamens. Scale bars are 1 cm in A and 1 mm in B. Additional file 3: Figure S3. Neighbor-joining phylogeny for *Coccinia* based on plastid DNA sequences. The concatenated *mat*K (605 bp) and *trn*S^GCU^-*trn*G^UCC^ intergenic spacer region (689 bp) were developed by editing and aligning the mentioned species sequences (Additional file 4: Table S1) in MEGA 5.1 software (Tamura K *et al*., [65]) with *Cucumis sativus* as an outgroup. The numbers at the branches of the tree represent the bootstrap support from 500 replicates. Species names follow Holstein and Renner [37] except for the gynomonoecious (GyM) sexual form of *Coccinia grandis.*
Additional file 4: Table S1. List of accession numbers of the sequences of the species used in phylogenetic tree for both *mat*K and *trn*S^GCU^-*trn*G^UCC^ intergenic spacer. All other sequences are used from previous work of Holstein and Renner [37]. Additional file 5: Figure S4. Analysis of seed content in *Coccinia grandis*. The seeds from the respective fruits were washed, counted and weighed for evaluating seed production per fruit. (A) Graphical representation of weight of seeds per fruit of female and gynomonoecious (GyM) plants. In the graph, the means ± s.e. are reported (*P <0.05, *t*-test); n = 10. (B) Graphical representation of average number of seeds per fruit of female and gynomonoecious (GyM) plants. In the graph, the means ± s.e. are reported (*P <0.05, *t*-test); n = 10. Additional file 6: Figure S5. Flower development in *Coccinia grandis*. Developmental stages of the flowers are assigned according to the length of the flower buds. (A) Male, (B) female and (C) gynomonoecious (GyM) flower buds. Scale bars =1 cm. Additional file 7: Figure S6. Longitudinal sections (L.S) of staminate flower buds of male plant showing pollen development. (A) and (B) are the sections of staminate flower of stages 8 and 12 respectively. p: Petals, st: stamens, pg: pollen grains. Scale bars are 2 mm. Additional file 8: Table S2. Sex modification in pistillate flower of *Coccinia grandis* female plant after treatment with different doses of silver nitrate. Additional file 9: Figure S7. Effects of silver nitrate (AgNO_3_) solution on flower development of gynomonoecious (GyM) plant. (A-D) Longitudinal sections of flowers at different developmental stages from silver nitrate treated gynomonoecious (GyM) plant (after spraying of 35 mM silver nitrate solution). p, petals; s, sepals; c, carpels; st, stamens; rst, rudimentary stamens; o, ovary. Scale bars are 1 cm in A; 2 mm in B,C and D. Additional file 10: Figure S8. Gynomonoecious *Coccinia grandis* with female and hermaphrodite flowers. (Herbarium Voucher: TU Campus, Karmakar, 433). Additional file 11: Table S3. List of primers. Additional file 12: Table S4. List of accession numbers of the sequences of the species used for designing degenerate primers.
