# Supplementary material for: Flower development, pollen fertility and sex expression analyses of three sexual phenotypes of Coccinia grandis
Source: BMC Plant Biol. 2014 Nov 28;14:325. doi: 10.1186/s12870-014-0325-0 (PMC4255441; doi:10.1186/s12870-014-0325-0)
Supplement: Additional file 1: Figure S1. — Floral phenotypes on gynomonoecious (GyM) plant. GyM plant showing both hermaphrodite (GyM–H) and pistillate (GyM–F) flowers on the same twig. [file 12870_2014_325_MOESM1_ESM.pdf]

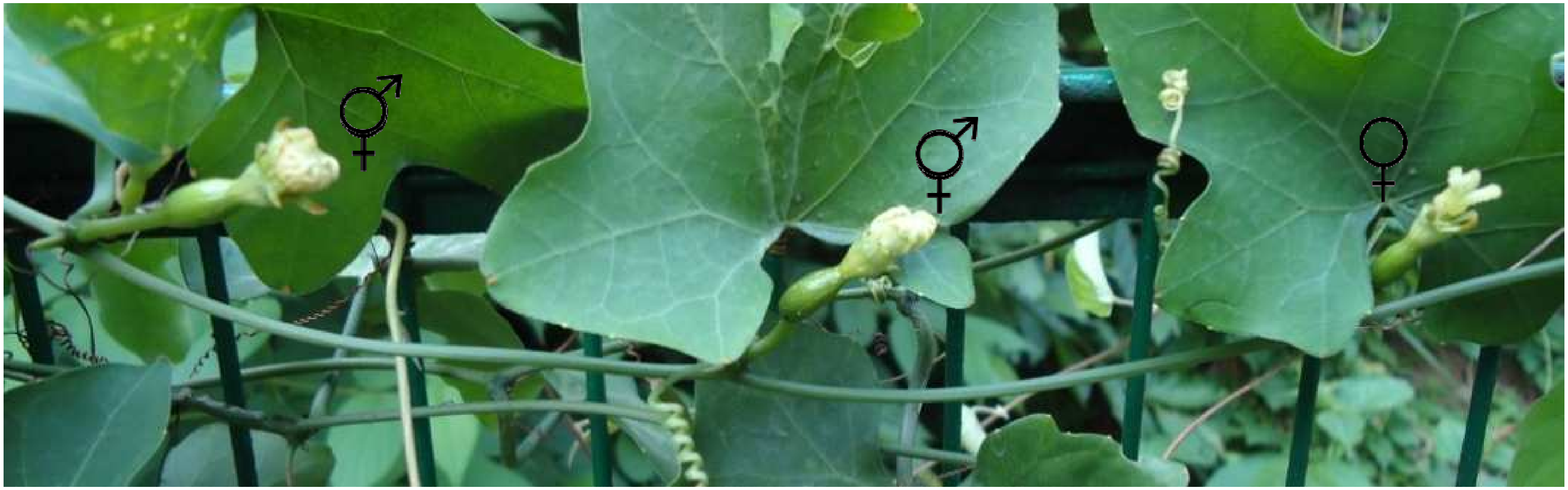

**Figure S1.** Floral phenotypes on gynomonoecious (GyM) plant. GyM plant showing both hermaphrodite (GyM–H) and pistillate (GyM–F) flowers on the same twig.
