# Supplementary material for: Flower development, pollen fertility and sex expression analyses of three sexual phenotypes of Coccinia grandis
Source: BMC Plant Biol. 2014 Nov 28;14:325. doi: 10.1186/s12870-014-0325-0 (PMC4255441; doi:10.1186/s12870-014-0325-0)
Supplement: Additional file 2: Figure S2. — Morphology of hermaphrodite (GyM–H) flowers of gynomonoecious (GyM) plant. Mature hermaphrodite (GyM-H) flower of gynomonoecious (GyM) showing incomplete development of stamens (A) and petaloid stamens (C). Longitudinal sections of early developmental stage of hermaphrodite (GyM–H) flower of gynomonoecious (GyM) plant (B). p: petals, s: sepals, c: carpels, st: stamens, rst: rudimentary stamens, o: ovary, pst: petaloid stamens. Scale bars are 1 cm in A and 1 mm in B. [file 12870_2014_325_MOESM2_ESM.pdf]

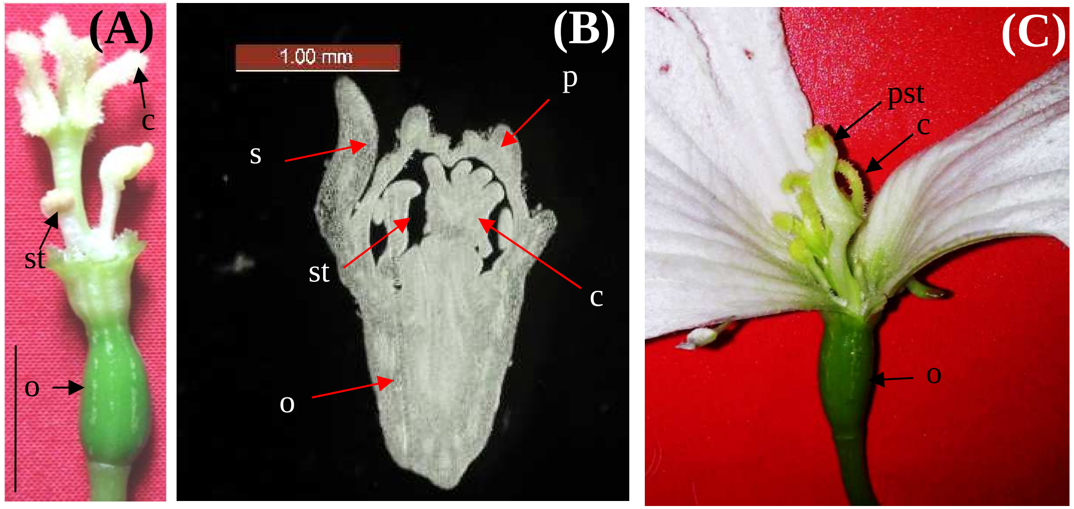

Figure S2. Morphology of hermaphrodite (GyM–H) flowers of gynomonoecious (GyM) plant. Mature hermaphrodite (GyM-H) flower of gynomonoecious (GyM) showing incomplete development of stamens (A) and petaloid stamens (C). Longitudinal sections of early developmental stage of hermaphrodite (GyM–H) flower of gynomonoecious (GyM) plant (B) . p, petals; s, sepals; c, carpels; st, stamens; rst, rudimentary stamens; o, ovary; pst, petaloid stamens. Scale bars are 1cm in A and 1mm in B.
