# Supplementary material for: Flower development, pollen fertility and sex expression analyses of three sexual phenotypes of Coccinia grandis
Source: BMC Plant Biol. 2014 Nov 28;14:325. doi: 10.1186/s12870-014-0325-0 (PMC4255441; doi:10.1186/s12870-014-0325-0)

Figure S3. Neighbor-joining phylogeny for *Coccinia* based on plastid DNA sequences. The concatenated *mat*K (605bp) and *trn*S^{GCU}-*trn*G^{UCC} intergenic spacer region (689bp) were developed by editing and aligning the mentioned species sequences (Table S1) in MEGA 5.1 software (Tamura K *et al*., 2011) with *Cucumis sativus* as an outgroup. The numbers at the branches of the tree represent the bootstrap support from 500 replicates. Species names follow Holstein and Renner (2011) except for the gynomonoecious (GyM) sexual form of *Coccinia grandis*.

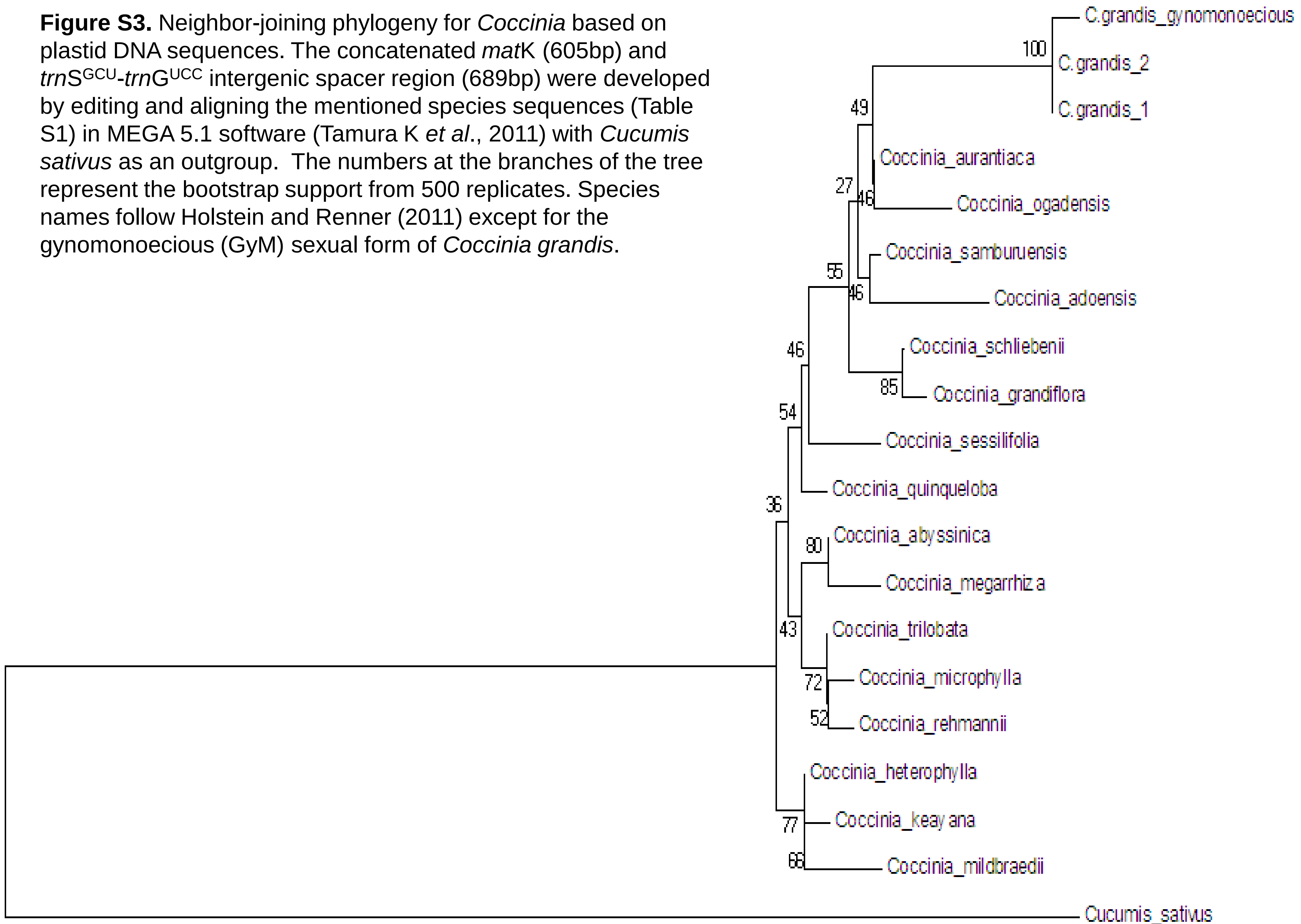

Supplement: Additional file 3: Figure S3. — Neighbor-joining phylogeny for Coccinia based on plastid DNA sequences. The concatenated matK (605 bp) and trnSGCU-trnGUCC intergenic spacer region (689 bp) were developed by editing and aligning the mentioned species sequences (Additional file 4: Table S1) in MEGA 5.1 software (Tamura K et al., [65]) with Cucumis sativus as an outgroup. The numbers at the branches of the tree represent the bootstrap support from 500 replicates. Species names follow Holstein and Renner [37] except for the gynomonoecious (GyM) sexual form of Coccinia grandis. [file 12870_2014_325_MOESM3_ESM.pdf]
