# Supplementary material for: Flower development, pollen fertility and sex expression analyses of three sexual phenotypes of Coccinia grandis
Source: BMC Plant Biol. 2014 Nov 28;14:325. doi: 10.1186/s12870-014-0325-0 (PMC4255441; doi:10.1186/s12870-014-0325-0)
Supplement: Additional file 4: Table S1. — List of accession numbers of the sequences of the species used in phylogenetic tree for both matK and trnSGCU-trnGUCC intergenic spacer. All other sequences are used from previous work of Holstein and Renner [37]. [file 12870_2014_325_MOESM4_ESM.pdf]

Table S1. List of accession numbers of the sequences used in phylogenetic tree for both *mat*K and *trn*S^{GCU}-*trn*G^{UCC} intergenic spacer. All other sequences are used from previous work of Holstein and Renner (2011).

**Species**	**Accession number**	
	***mat*K**	***trn*S^{GCU}-*trn*G^{UCC} intergenic spacer**
Coccinia grandis GyM	AB859710	AB859713
Coccinia grandis 1	AB859708	AB859711
Coccinia grandis 2	AB859709	AB859712
