# Supplementary material for: Flower development, pollen fertility and sex expression analyses of three sexual phenotypes of Coccinia grandis
Source: BMC Plant Biol. 2014 Nov 28;14:325. doi: 10.1186/s12870-014-0325-0 (PMC4255441; doi:10.1186/s12870-014-0325-0)
Supplement: Additional file 5: Figure S4. — Analysis of seed content in Coccinia grandis. The seeds from the respective fruits were washed, counted and weighed for evaluating seed production per fruit. (A) Graphical representation of weight of seeds per fruit of female and gynomonoecious (GyM) plants. In the graph, the means ± s.e. are reported (*P <0.05, t-test); n = 10. (B) Graphical representation of average number of seeds per fruit of female and gynomonoecious (GyM) plants. In the graph, the means ± s.e. are reported (*P <0.05, t-test); n = 10. [file 12870_2014_325_MOESM5_ESM.pdf]

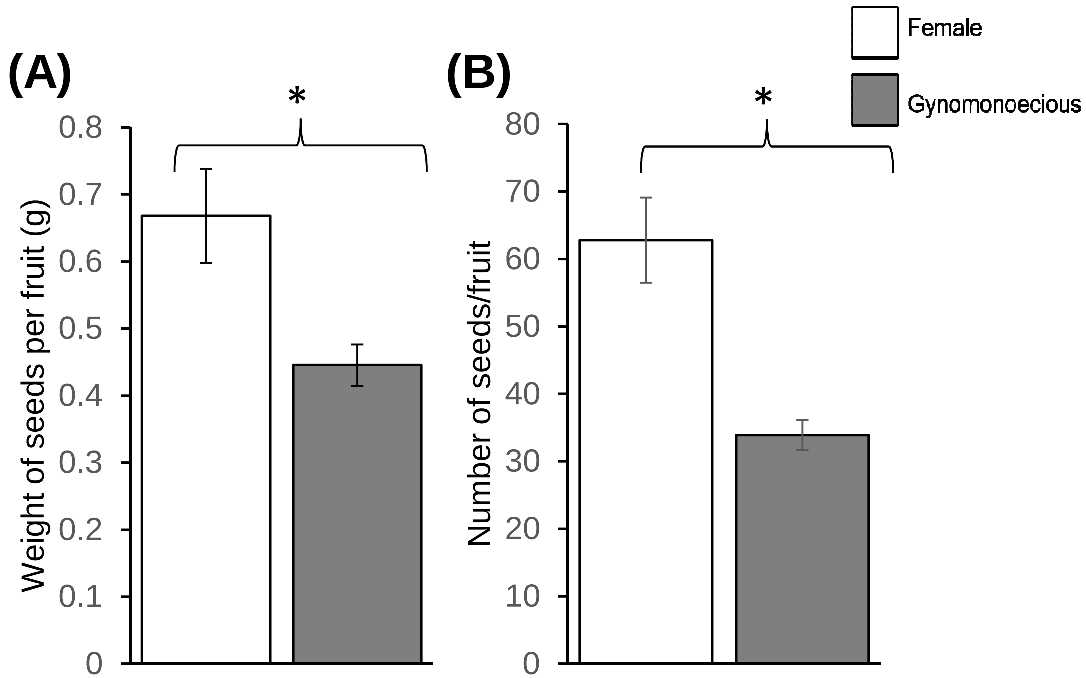

Figure S4. Analysis of seed content in *Coccinia grandis*. The seeds from the respective fruits were washed, counted and weighed for evaluating seed production per fruit. (A) Graphical representation of weight of seeds per fruit of female and gynomonoecious (GyM) plants. In the graph, the means ± s.e. are reported (*P < 0.05, *t*-test); n=10. (B) Graphical representation of average number of seeds per fruit of female and gynomonoecious (GyM) plants. In the graph, the means ± s.e. are reported (*P < 0.05, *t*-test); n=10.
