# Supplementary material for: Flower development, pollen fertility and sex expression analyses of three sexual phenotypes of Coccinia grandis
Source: BMC Plant Biol. 2014 Nov 28;14:325. doi: 10.1186/s12870-014-0325-0 (PMC4255441; doi:10.1186/s12870-014-0325-0)
Supplement: Additional file 6: Figure S5. — Flower development in Coccinia grandis. Developmental stages of the flowers are assigned according to the length of the flower buds. (A) Male, (B) female and (C) gynomonoecious (GyM) flower buds. Scale bars =1 cm. [file 12870_2014_325_MOESM6_ESM.pdf]

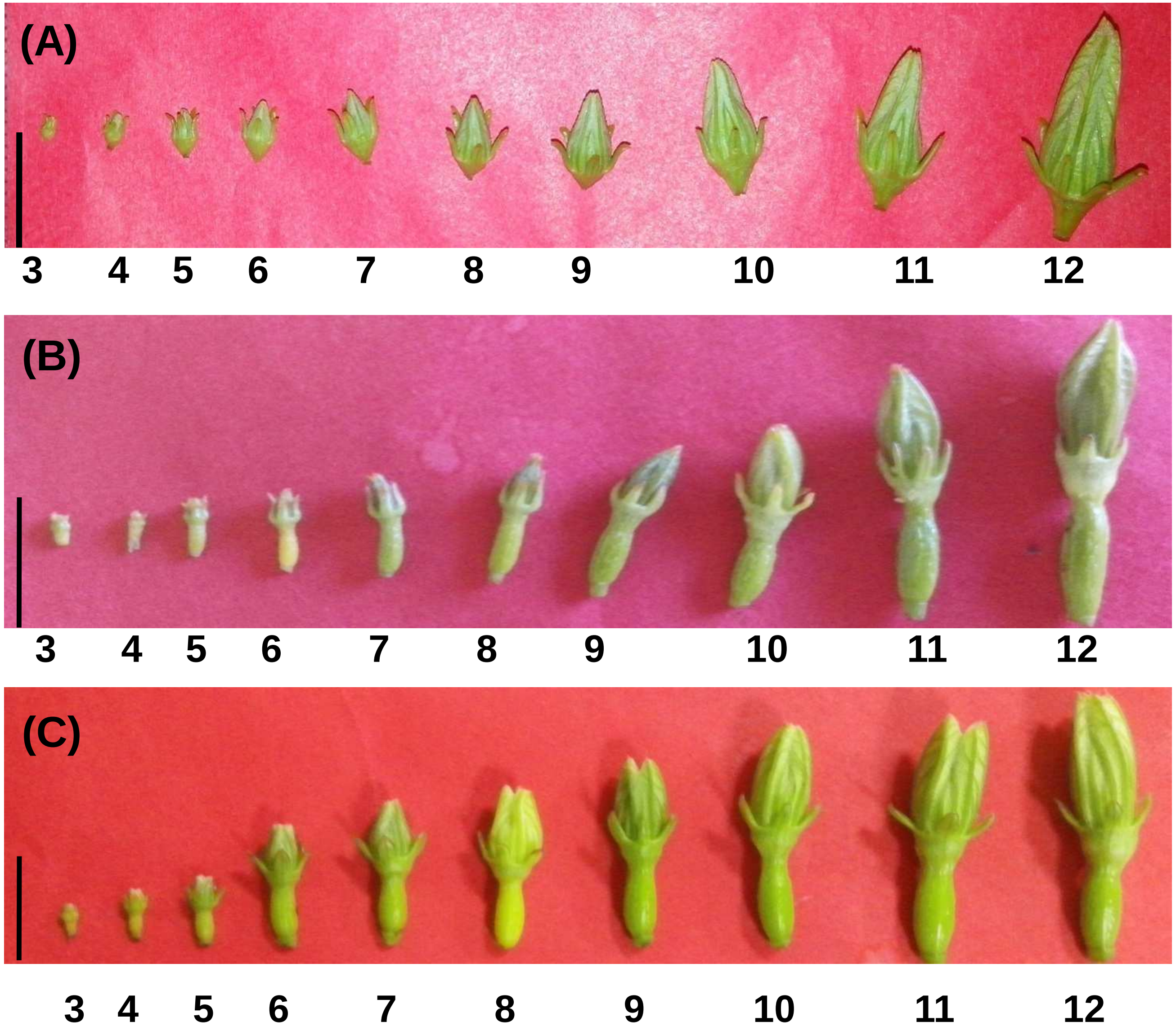

Figure S5. Flower development in *Coccinia grandis*. Developmental stages of the flowers are assigned according to the length of the flower. buds. (A) Male, (B) female and (C) gynomonoecious (GyM) flower buds. Scale bars = 1 cm.
