# Supplementary material for: Flower development, pollen fertility and sex expression analyses of three sexual phenotypes of Coccinia grandis
Source: BMC Plant Biol. 2014 Nov 28;14:325. doi: 10.1186/s12870-014-0325-0 (PMC4255441; doi:10.1186/s12870-014-0325-0)
Supplement: Additional file 7: Figure S6. — Longitudinal sections (L.S) of staminate flower buds of male plant showing pollen development. (A) and (B) are the sections of staminate flower of stages 8 and 12 respectively. p: Petals, st: stamens, pg: pollen grains. Scale bars are 2 mm. [file 12870_2014_325_MOESM7_ESM.pdf]

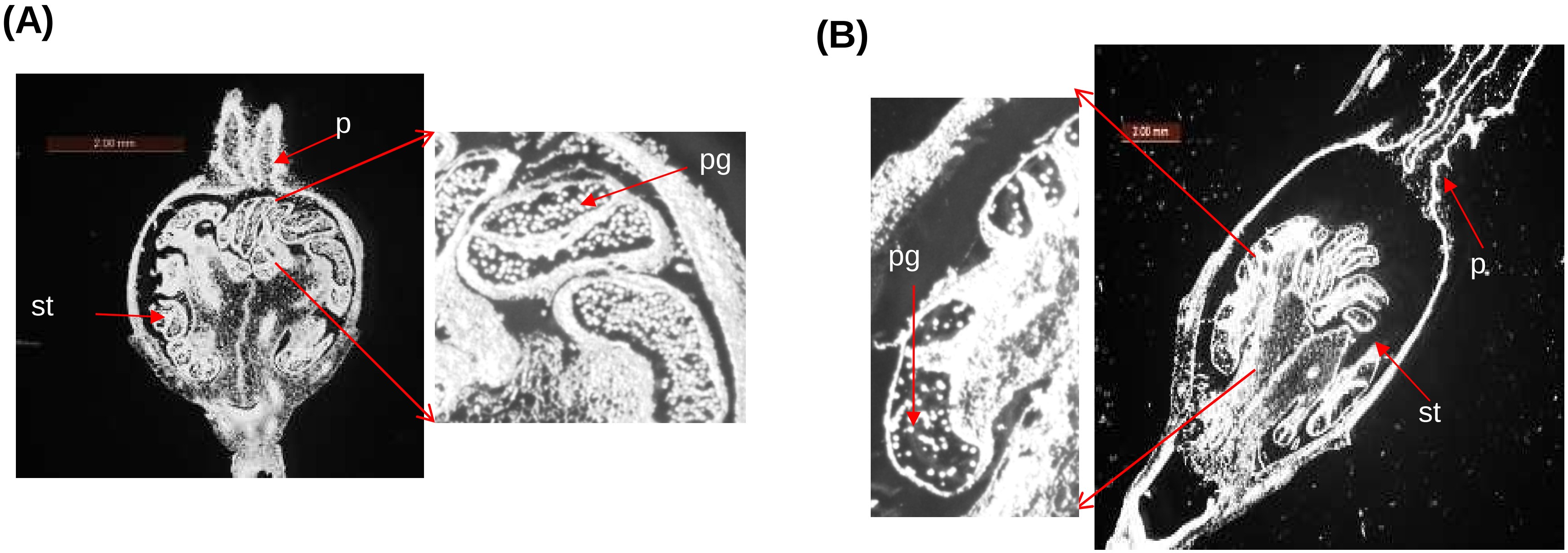

Figure S6. Longitudinal sections (L.S) of staminate flower buds of male plant showing pollen development. (A) and (B) are the sections of staminate flower of stages 8 and 12 respectively. p: Petals, st: stamens, pg: pollen grains. Scale bars are 2mm.
