# Supplementary material for: Flower development, pollen fertility and sex expression analyses of three sexual phenotypes of Coccinia grandis
Source: BMC Plant Biol. 2014 Nov 28;14:325. doi: 10.1186/s12870-014-0325-0 (PMC4255441; doi:10.1186/s12870-014-0325-0)
Supplement: Additional file 8: Table S2. — Sex modification in pistillate flower of Coccinia grandis female plant after treatment with different doses of silver nitrate. [file 12870_2014_325_MOESM8_ESM.pdf]

Table S2. Sex modification in pistillate flower of *Coccinia grandis* female plant after treatment with different doses of silver nitrate.

$AgNO_3$ conc. tested	No. of sprays (7 days apart)	Effect	Days required for modification of sex after first spray	Days required for reversion to normal pistillate flower after second spray
20 mM	2-3	Incomplete development of stamens from staminodes. Ectopic expression also occurs.	15 – 20 days	10 - 12 days
25 mM	2-3	Staminodes converts into stamens. Rare complete sex modification is also observed after 3 exposures.	15 - 20 days	10 - 12 days
30 mM	1-2	Complete conversion of staminodes into stamens (100 %).	10-12 days	15 – 18 days
35 mM	1	Complete conversion of staminodes into stamens (100 %).	10-12 days	12- 15 days
>35 mM	1	Lethal	-	-
