# Supplementary material for: Flower development, pollen fertility and sex expression analyses of three sexual phenotypes of Coccinia grandis
Source: BMC Plant Biol. 2014 Nov 28;14:325. doi: 10.1186/s12870-014-0325-0 (PMC4255441; doi:10.1186/s12870-014-0325-0)
Supplement: Additional file 9: Figure S7. — Effects of silver nitrate (AgNO3) solution on flower development of gynomonoecious (GyM) plant. (A-D) Longitudinal sections of flowers at different developmental stages from silver nitrate treated gynomonoecious (GyM) plant (after spraying of 35 mM silver nitrate solution). p, petals; s, sepals; c, carpels; st, stamens; rst, rudimentary stamens; o, ovary. Scale bars are 1 cm in A; 2 mm in B,C and D. [file 12870_2014_325_MOESM9_ESM.pdf]

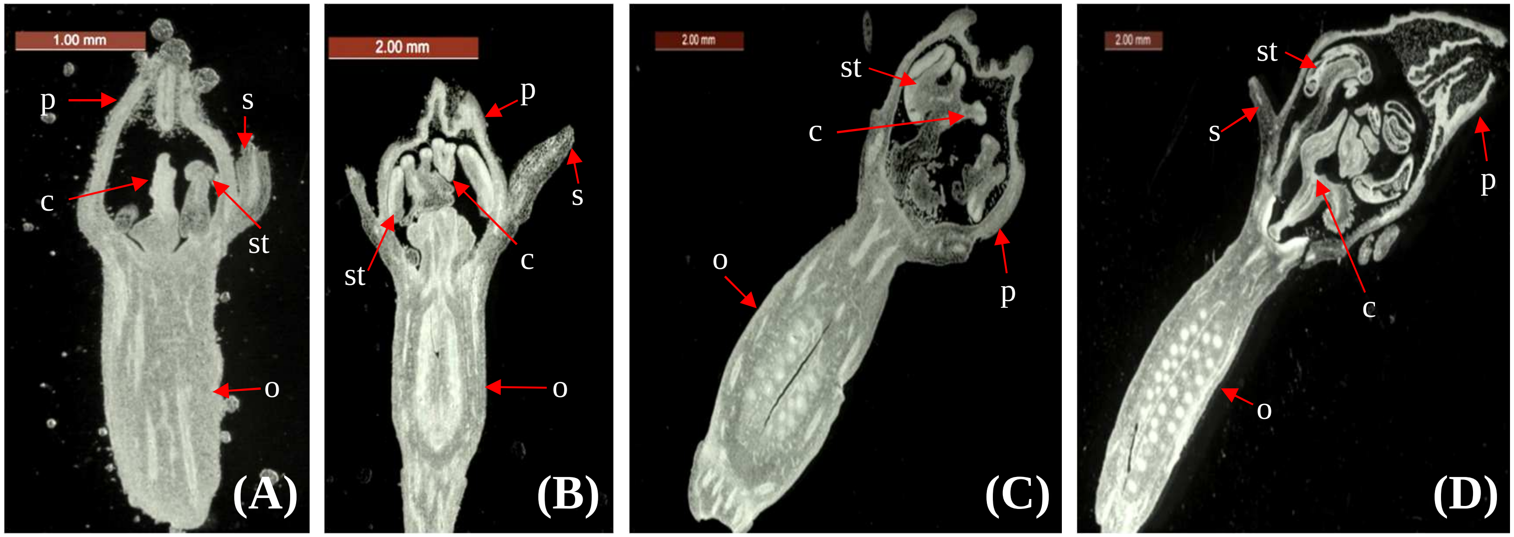

Figure S7. Effects of silver nitrate ($AgNO_3$) solution on flower development of gynomonoecious (GyM) plant. (A-D) Longitudinal sections of flowers at different developmental stages from silver nitrate treated gynomonoecious (GyM) plant (after spraying of 35 mM silver nitrate solution). p, petals; s, sepals; c, carpels; st, stamens; rst, rudimentary stamens; o, ovary. Scale bars are 1cm in A; 2mm in B,C and D.
