# Supplementary material for: Flower development, pollen fertility and sex expression analyses of three sexual phenotypes of Coccinia grandis
Source: BMC Plant Biol. 2014 Nov 28;14:325. doi: 10.1186/s12870-014-0325-0 (PMC4255441; doi:10.1186/s12870-014-0325-0)
Supplement: Additional file 10: Figure S8. — Gynomonoecious Coccinia grandis with female and hermaphrodite flowers. (Herbarium Voucher: TU Campus, Karmakar, 433). [file 12870_2014_325_MOESM10_ESM.pdf]

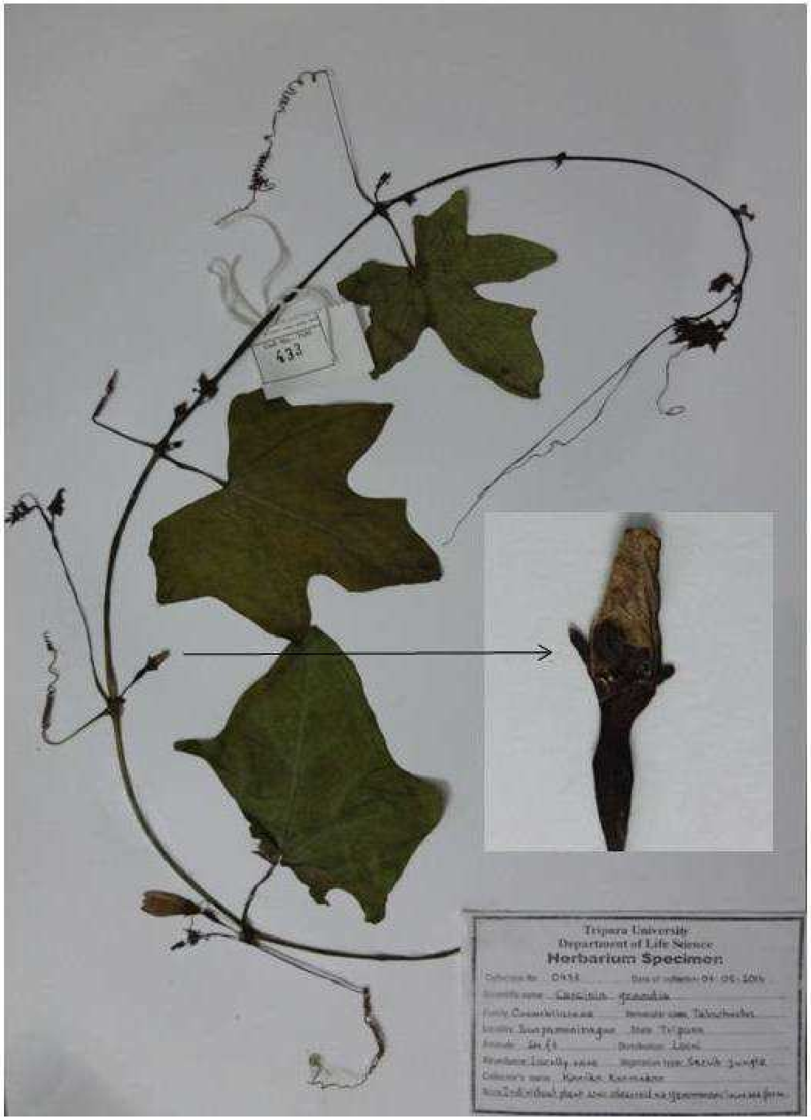

Figure S8. Gynomonoecious *Coccinia grandis* with female and hermaphrodite flowers. (Herbarium Voucher: TU Campus, Karmakar, 433)
