# Supplementary material for: Flower development, pollen fertility and sex expression analyses of three sexual phenotypes of Coccinia grandis
Source: BMC Plant Biol. 2014 Nov 28;14:325. doi: 10.1186/s12870-014-0325-0 (PMC4255441; doi:10.1186/s12870-014-0325-0)
Supplement: Additional file 11: Table S3. — List of primers. [file 12870_2014_325_MOESM11_ESM.pdf]

Table S3. List of primers.

	Primer	Primer sequence
DNA barcoding (Hamilton, 1999)	matK Forward	5’- ATCCATCTGGAAATCTTAGTTC-3’
	matK Reverse	5’- CTTCCTCTGTAAAGAATTC-3’
	trnS Forward	5’- GCCGCTTTAGTCCACTCAGC-3’
	trnG Reverse	5’- GAACGAATCACACTTTTACCAC-3’
RACE Primers	PI GSP1 (5’ RACE)	5’-TGGTTGGATCGGTTGCACTCTGAAGGC-3’
	PI GSP2 (3’ RACE)	5’-TCACTGGTGTTCGTGAGAAGCAGTCGGAG-3’
	AG GSP1 (3’ RACE)	5’-TCTATGTGATGCTGAAGTTGCTCTAATCG-3’
	AG GSP2 (5’RACE)	5’-TAGTGGAATCTGAGGATGCCTTCTTGTATC-3’
Gene Identification	CgPI B12 F	5’-GGAAAAAGACTGTGGGATGCNAARCAYGA-3’
	CgPI D8 R	5’-TCTTTCTTGCAGATTTGGTTGNATNGGYTG-3’
	CgAG A26 F	5’-GAGGAAAGATTGAAATTAAGAGAATHGARAAYAC-3’
	CgAG A51 R	5’-CTCTCAGCTTAGCAGCTTCYTGYTGRTA-3’
qRT-PCR and cDNA synthesis	CgPI RTB-F	5’ - GGGAAGAGGCTGTGGGATGCTAAG - 3’
	CgPI RTB-R	5’ - CTCCGACTGCTTCTCACGAACACC - 3’
	CgAG RTC-F	5’ - GAGGCCAGATTGGAGAGAGGAATCAG - 3’
	CgAG RTC-R	5’ - GTTTGTTGTTGTTGCTGCTGCTGTTG - 3’
	18S-FP2	5’- GGGCATTCGTATTTCATAGTCAGAG - 3’
	18S-R	5’ -CGGTTCTTGATTAATGAAAACATCCT - 3’
