# Supplementary material for: Flower development, pollen fertility and sex expression analyses of three sexual phenotypes of Coccinia grandis
Source: BMC Plant Biol. 2014 Nov 28;14:325. doi: 10.1186/s12870-014-0325-0 (PMC4255441; doi:10.1186/s12870-014-0325-0)
Supplement: Additional file 12: Table S4. — List of accession numbers of the sequences of the species used for designing degenerate primers. [file 12870_2014_325_MOESM12_ESM.pdf]

Table S4. List of accession numbers of the sequences of the species used for designing degenerate primers.

AGAMOUS and homologs		PISTILLATA and homologs	
**Accession No.**	**Species**	**Accession No.**	**Species**
ACH72974.1	*Prunus serotina*	AAD02250.1	*Cucumis sativus*
ABA39727.1	*Theobroma cacoa*	AAS46018.1	*Petunia hybrida*
CAC80858.1	*Malus domestica*	AAY79173.1	*Vitis vinifera*
AAC08528.1	*Cucumis sativus*	ABQ51323.1	*Carica papaya*
ABC25564.1	*Momordica charantia*	ABS32248.1	*Prunus persica*
AEU08497.1	*Corylus heterophylla*	ADU15475.1	*Actinidia chinensis*
XP_002283924.1	*Vitis vinifera*	AED92817.1	*Arabidopsis thaliana*
Q40885.1	*Petunia hybrida*	AER30449.1	*Passiflora edulis*
CAJ44130.1	*Misopates orontium*	CAC28021.1	*Malus domestica*
CAA16753.1	*Arabidopsis thaliana*	CAD32764.1	*Betula pendula*
